# Beyond intelligence: Exploring the role of growth mindsets in the domain of social–emotional skills

**DOI:** 10.1111/bjep.70037

**Published:** 2025-10-02

**Authors:** Jianhua Zhang, Faming Wang, Ronnel B. King

**Affiliations:** ^1^ Department of Curriculum and Instruction The Chinese University of Hong Kong Hong Kong SAR China; ^2^ College of Education Zhejiang University Hangzhou China

**Keywords:** domain specificity, growth mindset, social–emotional skills

## Abstract

**Background:**

Growth mindsets refer to the belief that personal attributes can be developed and improved through learning and effort. Much of the prior work on mindsets has focused on mindsets of intelligence, with little attention devoted to whether and how growth mindsets might also be relevant to the domain of social–emotional skills.

**Aims:**

To address this gap, this study aimed to extend research on growth mindsets to the domain of social–emotional skills and examine the associations between growth mindsets and various types of social–emotional skills. We explored five broad social–emotional skills and 15 specific facet‐level skills, including task performance (self‐control, responsibility, persistence), emotional regulation (stress resistance, emotional control, optimism), engaging with others (energy, assertiveness, sociability), collaboration (empathy, cooperation, trust) and open mindedness (curiosity, creativity, tolerance).

**Sample:**

We drew on data from 29,798 fifteen‐year‐old students from 10 cities across nine countries.

**Methods:**

Hierarchical linear modelling was employed to investigate the association between growth mindsets and various types of social–emotional skills.

**Results:**

Results indicated that a growth mindset of social–emotional skills was positively associated with five broad social–emotional skills: task performance, emotional regulation, engaging with others, collaboration, and open‐mindedness. These results also applied to the 15 specific facet‐level skills. Interestingly, we also found that mindsets of social–emotional skills were most strongly associated with emotional regulation.

**Conclusion:**

This study extended the growth mindset literature by applying it to the domain of social–emotional skills. Our findings may have promising implications for future interventions aimed at improving students' social–emotional skills.

## INTRODUCTION

Students with a growth mindset believe that personal characteristics can be developed (Dweck & Leggett, 1988). Much of the work on mindsets has primarily focused on intelligence mindsets, which pertain to one's beliefs about the malleability of intelligence (Dweck & Yeager, 2019). These studies have demonstrated the critical role of growth mindsets in students' academic functioning (Dweck & Leggett, 1988; Yeager & Dweck, 2020). Students who hold a growth mindset of intelligence are more likely to have optimal academic outcomes, such as setting mastery‐oriented goals (DeBacker et al., 2018), exhibiting positive responses to academic failure (Dweck & Yeager, 2019), and achieving higher academic performance (Yeager et al., 2019). By contrast, individuals with a fixed mindset of intelligence are more likely to avoid challenges and fail to reach their full potential (Dweck & Yeager, 2021).

Historically, prior work has primarily focused on mindsets of intelligence and their impact on students' academic outcomes (Yeager & Dweck, 2020). More recent developments, however, have extended implicit theories to mindsets of more transient and malleable states, such as thoughts, feelings and behaviours (Schleider & Weisz, 2016a, 2016b). For example, studies have investigated the importance of growth mindsets in the domain of mental health (Yeager & Dweck, 2023), stress (Crum et al., 2020) and emotions (King & Dela Rosa, 2019; Somerville et al., 2024; Tamir et al., 2007). They are closer to changeable states and behaviours than to entrenched personality traits. Therefore, mindsets of states may be a more direct and potent predictor than broader mindsets of global traits (Schleider & Weisz, 2016a). Prior studies also suggest that domain‐specific mindsets are more closely associated with the corresponding outcomes than mindsets of intelligence (Schroder et al., 2016), justifying the need to explore domain‐specific growth mindsets.

Given the importance of mindsets across various domains, an important question is whether growth mindsets are relevant to the domain of social–emotional skills. Social–emotional skills have been found critical to key academic, psychological and life outcomes (Cipriano et al., 2023). Many studies have explored the role of social contexts in developing social–emotional skills; however, little is known about how motivational factors are associated with these skills. Just as individuals hold different beliefs about the malleability of their intelligence, might it be possible that students also hold different beliefs about the malleability of their social–emotional skills? Growth mindsets of social–emotional skills (perceiving social–emotional skills as malleable and changeable through effort) may guide how individuals interpret and respond to emotional processes and social interactions, such as adopting adaptive emotional regulation strategies and employing positive coping strategies in the face of social setbacks. Therefore, this study aimed to explore the role played by growth mindsets in students' social–emotional skills.

## LITERATURE REVIEW

### Growth mindset of intelligence and domain‐specific mindsets

The concept of mindsets emerged from work on implicit theories about whether human qualities (e.g. intelligence) are changeable or fixed, which corresponds to ‘growth mindset’ and ‘fixed mindset’, respectively (Dweck & Leggett, 1988). A growth mindset refers to the belief that attributes, such as intelligence, are malleable and can be changed through individual effort (Dweck, 2006). These beliefs enable students to exert more effort towards academic learning, persist longer in challenging tasks and employ more adaptive self‐regulated strategies, which in turn benefit school success (Dweck & Yeager, 2019). By contrast, a fixed mindset assumes that personal attributes cannot be changed, which could undermine learning (Dweck, 2006).

Research on mindsets has mostly focused on the intelligence domain, with studies showing positive associations between growth mindsets of intelligence and academic outcomes (Blackwell et al., 2007; King & Trinidad, 2021). For example, in a meta‐analysis of 273 studies, Sisk et al. (2018) identified a positive correlation between growth mindsets and academic achievement (*r* = .10). More recently, Burnette et al. (2023) conducted a systematic review and meta‐analysis and revealed a positive association between growth mindset interventions and academic achievement based on 53 independent samples (Cohen's *d* = .14).

Aside from intelligence mindsets, researchers have also called for the need to explore domain‐specific mindsets (Yeager et al., 2022). Mindsets in a specific domain are presumed to be associated with outcomes in the same domain (Dweck, 1996). Dweck and colleagues maintained that ‘at the assessment level, endorsing an entity theory of one attribute is statistically independent of endorsing an entity theory of a different attribute’ (Dweck et al., 1995, p. 269). For example, a student may hold a growth mindset of her mathematics ability, which could motivate her to delve into her math schoolwork, but this does not necessarily translate into the language learning domain (Bui et al., 2023; Heyder et al., 2021). A comprehensive review comprising 125 studies indicated that growth mindsets of conceptually distinct domains (e.g. intelligence and social–emotional skills) are only weakly correlated with each other (Kyler & Moscicki, 2024). This makes it especially important to explore the mindsets most relevant to a particular domain.

Studies found that domain‐specific mindsets are more predictive of outcomes within their corresponding domain than in unrelated domains. For example, a meta‐analytic study found that psychological distress showed a higher correlation with mindsets of emotion (*r* = −.29) than mindsets of intelligence (*r* = −.11) (Burnette et al., 2020). Tamir et al. (2007) found that emotion mindsets had a larger effect on emotional regulation than intelligence mindsets. More recently, Yeager et al. (2022) found that a stress mindset had a larger effect on stress than an intelligence mindset. These studies showed the importance of domain‐matching, as mindsets in the relevant domain are more likely to shape functioning within that domain.

### Big Five theory: Social–emotional skills

Social–emotional skills are defined as ‘individual capacities that can be (a) manifested in consistent patterns of thoughts, feelings and behaviours; (b) developed through formal and informal learning experiences; and (c) important drivers of socioeconomic outcomes throughout the individual's life’ (OECD, 2015, p. 35). Although different frameworks have been used to describe social–emotional skills, their key areas of overlap resemble the Big Five taxonomy (e.g., Collaborative for Academic, Social, and Emotional Learning, 2020; Collie & Martin, 2024). The Big Five framework integrates a wide range of social–emotional characteristics (Soto et al., 2021). Additionally, the Big Five framework articulates more specific, facet‐level constructs, representing hierarchical constructs of social–emotional skills.

It is important to note that social–emotional skills are distinct from personality traits (Soto et al., 2022). Personality traits are defined as broad and stable characteristics that represent how someone tends to think, feel and behave, averaged across various situations and contexts (Soto et al., 2021, 2024). Skills are more malleable and context‐dependent. They are also more likely to be developed through experience and learning. Social–emotional skills represent an individual's ability to think, feel and behave appropriately when the situation calls for it.

Social–emotional skills include five domains corresponding to the Big Five and 15 specific facets:

*Task performance (resembling conscientiousness)*: The ability to be self‐controlled, responsible, and persistent, including *self‐control* (control impulses, delay gratification and maintain concentration), *responsibility* (follow promises to others), and *persistence* (persevere in tasks and activities despite challenges and distractions).

*Emotional regulation (resembling emotional stability)*: The ability to manage and control negative emotional responses and be optimistic, including *stress resistance* (effectiveness in modulating anxiety and response to stress), *emotional control* (keeping one's emotions and temper under control), and *optimism* (positive expectations for self and life).

*Engaging with others (resembling extraversion)*: The ability to engage and enjoy others' company, including *energy* (sustaining vigorous activity), *assertiveness* (enjoying leadership, dominance and assertive behaviour), and *sociability* (preference for social interactions).

*Collaboration (resembling agreeableness)*: The ability to collaborate successfully with others by maintaining positive relations and being sympathetic to others, including *empathy* (perspective taking and empathic concern for others' well‐being), *cooperation* (living in harmony with others and valuing interconnectedness among all people), and *trust* (assuming that others have good intentions).

*Open‐mindedness (resembling openness)*: The ability to generate new ideas and keep an open mind, including *curiosity* (interest in ideas and love of learning, and intellectual exploration), *creativity* (generating novel ideas or products), and *tolerance* (open to different points of view, values diversity).

Social–emotional skills have been found to be positively associated with academic success (Corcoran et al., 2018; Wigelsworth et al., 2022). Given the importance of social–emotional skills, an increasing number of studies are exploring the factors that may shape these skills (e.g. Mahoney et al., 2021; Wang et al., 2024; Wang et al., 2025). More recently, researchers have started to consider motivational factors (e.g. growth mindsets) in the domain of social–emotional skills (Collie, 2021).

### Beyond intelligence: Growth mindsets of social–emotional skills

A growth mindset of social–emotional skills refers to the belief that social–emotional skills (e.g. emotional regulation, social skills) are malleable and controllable and can be changed through effort and persistence (Ford & Gross, 2019; Yeager et al., 2014). When students adopt a growth mindset regarding their social–emotional skills, they are more likely to develop adaptive social and emotional strategies. Although direct evidence related to mindsets of social–emotional skills is still developing, several studies have shown the critical role of growth mindsets in domains closely related to social–emotional skills (e.g. emotion controllability mindsets; Ford & Gross, 2018, 2019).

Theoretically, a growth mindset of social–emotional skills could shape the type of emotion regulation strategies employed by the individual (Ford & Gross, 2018, 2019). For example, beliefs about the controllability of emotions may influence whether and how individuals draw on different emotional regulation stages, including identifying a need to regulate one's emotions, selecting strategies, implementing those strategies and monitoring their regulatory success. Students with a fixed mindset might be less likely to use adaptive emotion regulation strategies, leading to impaired emotional regulation (Kneeland et al., 2016). For example, Ford et al. (2018) found that individuals with more of a fixed mindset had greater depressive symptoms, which was accounted for by their lower propensity to engage in cognitive reappraisal strategies.

Additionally, students with a growth mindset of social–emotional skills might be more flexible in dealing with complex social situations (Yeager et al., 2014). For example, when facing challenging social situations (e.g. peer exclusion), students with a growth mindset tend to employ more positive coping strategies in the face of adversity (Yeager et al., 2014). On the contrary, if students hold a fixed mindset, they may become more stressed and resort to negative coping strategies, given their belief that they lack control over their social and emotional responses to adverse circumstances.

Taken together, social–emotional skill mindsets can guide how individuals interpret and respond to their own affective and interpersonal experiences. A growth mindset fosters the perception that social‐emotional skills are malleable and can be improved with effort and effective strategies. This belief is likely to promote adaptive self‐regulatory processes, such as greater attentional control, openness to feedback and persistence in the face of social setbacks. Conversely, a fixed mindset reinforces the notion that one's emotions or social abilities are static and unchangeable, which may constrain students' motivation to regulate emotions or engage constructively in social contexts. Over time, these mindsets can have cumulative effects on well‐being and adjustment.

### Accounting for the role of different covariates

To account for alternative explanations, student‐level and school‐level covariates were added to the model.

At the student level, a growth mindset of intelligence has been frequently studied (Burnette et al., 2023). Gender differences in social–emotional skills have also been examined. Socioeconomic status (SES) has been found to be important for social–emotional skills (Lee et al., 2024).

At the school level, school climate is critical for students' social–emotional skills (Goldberg et al., 2019; Wang et al., 2024). Class size has a positive impact on social–emotional skills (Coelho & Sousa, 2017). School policies that encourage the promotion of social–emotional skills might also play an important role in the development of students' social–emotional skills (Kankaraš & Suarez‐Alvarez, 2019). Hence, we included these covariates in the study.

### Cross‐cultural context

It is also helpful to paint the context for the current study. This study drew on the OECD Social and Emotional Skills Survey (SSES), which was conducted across nine countries (Colombia, Korea, Finland, United States, Turkey, Russian Federation, Canada, Portugal and China). Prior work on social–emotional skills has primarily drawn on one cultural context, mostly the United States. For example, Corcoran et al.'s (2018) review revealed that 97.5% of the studies were from the US. Hence, by drawing on a broader range of countries, this study extends the cross‐cultural generalizability of the results.

### The present study

The present study aimed to examine the association between growth mindsets and social–emotional skills. Specifically, we assessed growth mindsets of social–emotional skills and how they were associated with the five broad social–emotional skills. We assumed that growth mindsets might have varying degrees of association with the different social–emotional skills, but given the lack of prior studies, we did not posit specific hypotheses about which associations would be stronger than others. The following hypothesis was tested:

*A growth mindset of social–emotional skills would be positively associated with the five broad social–emotional skills (i.e. task performance, emotional regulation, engaging with others, collaboration and open‐mindedness)*.

**FIGURE 1:**
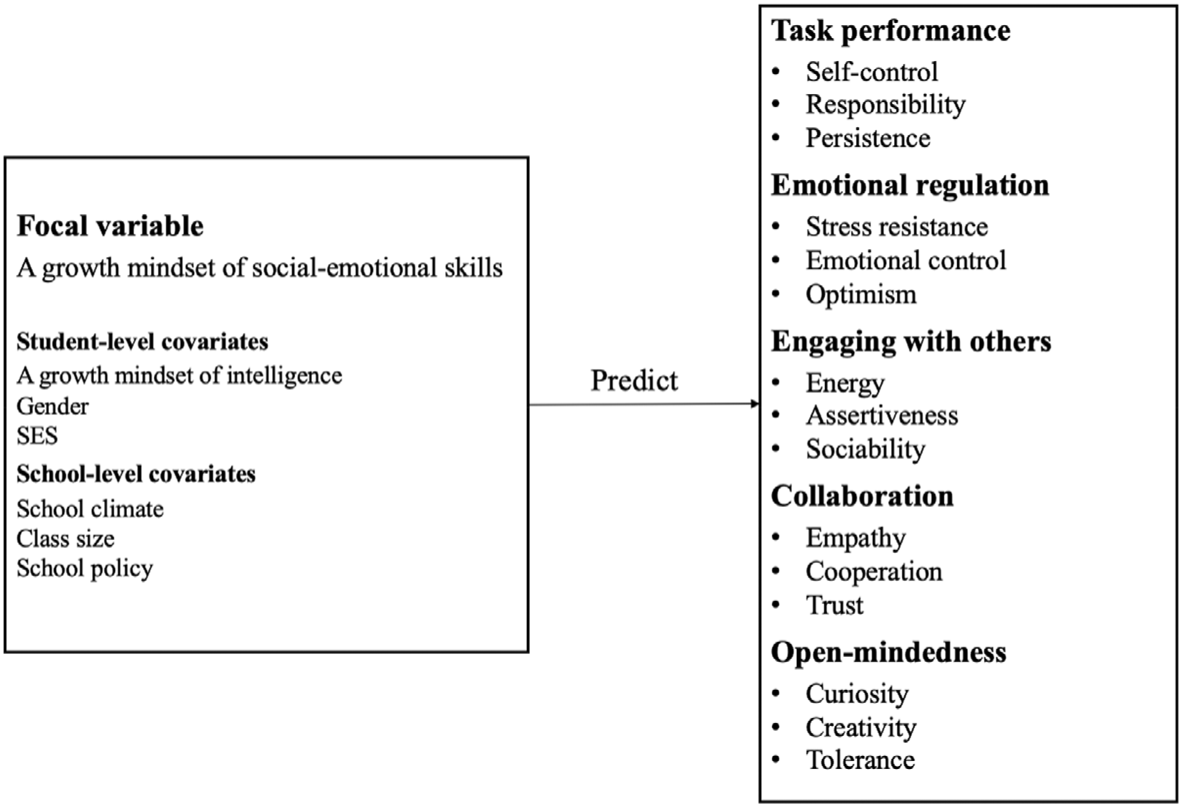
Conceptual model.

The conceptual framework for the present study is presented in Figure 1.

Although we recognize the SSES data set is cross‐sectional, which limits causal claims, there is a long line of research placing a growth mindset as a predictor of focal outcomes (e.g. Burnette et al., 2023). In this study, we model a growth mindset of social–emotional skills as a predictor of social–emotional skills. However, we revisit this issue and argue for due caution in our discussion of the study limitations.

## METHODS

### Participants

This study utilized data from the first round of the OECD SSES (https://www.oecd.org/education/ceri/social‐emotional‐skills‐study/data.htm). The data were collected in 2019, which includes 29,798 15‐year‐olds (51.24% girls). The OECD SSES is a large‐scale educational assessment programme that focuses on social–emotional skills. The programme collected data from 10 cities across nine countries. A complex two‐stage sampling design was employed in data collection to obtain a representative sample, where students are nested within schools.

### Measures

All variables were derived from the SSES database. The OECD has conducted a series of analyses based on item response theory and confirmatory factor analysis and showed high levels of reliability and construct validity across cities (OECD, 2021).^1^

#### Focal variable

The growth mindset of social–emotional skills was measured by two items (‘Your emotional skills are something about yourself that you cannot change very much’ and ‘Your social skills are something about yourself that you cannot change very much’, reverse coded; Cronbach's *α* = .80) on a 5‐point Likert Scale (1 = *Strongly disagree*, 5 = *Strongly agree*).

#### Outcome variables

The values of broad skills were aggregated from the scores on the facet‐level skills (Guo et al., 2023). Each item was rated on a 5‐point Likert scale (1 = *Strongly disagree*, 5 = *Strongly agree*). All the subscales of 15 facets have good reliability (Cronbach's *α* > .74). Sample items can be found in Table S1.

#### Covariates

A growth mindset of intelligence, gender, and SES was used as student‐level covariates. School‐level covariates included school climate, class size and school policy.

The growth mindset of intelligence was measured by one item on a 5‐point Likert scale (‘Your intelligence is something about yourself that you cannot change very much’, reverse coded; 1 = *Strongly disagree*, 5 = *Strongly agree*).^2^

SES was operationalized as economic, social and cultural status (ESCS) (Kankaraš & Suarez‐Alvarez, 2019).^3^

School climate was measured using a questionnaire administered to the principals. Principals were asked about the extent (‘*not at all*’, ‘*a little*’, *‘to some extent*’ or ‘*a lot*’) to which disruptive behaviours among students and teachers hindered student learning. These included dimensions such as students' disruptive behaviours (five items, e.g. students skipping classes; Cronbach's *α* = .89) and teachers' disruptive behaviours (six items, e.g. teachers not being well prepared for classes; Cronbach's *α* = .89).

Class size was measured by one item (What is the average size of classes in your school?).

School policy was measured using eight items (e.g. Teachers are asked to promote the development of students' social and emotional skills as part of their work; Cronbach's *α* = .67) with ‘Yes’ or ‘No’. ‘Yes’ was coded as 1, and ‘No’ was coded as 0. This variable was reported by principals.

### Analysis

All the analyses were conducted using Stata (Rabe‐Hesketh & Skrondal, 2008).

#### Missing data

Missing data range from .14% to 15.66% in this study. Maximum likelihood estimation was accomplished using the expectation maximization algorithm (Dempster et al., 1977).

#### Preliminary analysis

The mean, standard deviation and correlation of variables were calculated.

Before running the primary analysis, we tested measurement models of the social–emotional skills. Model fit was assessed by comparative fit index (*CFI*), Tucker‐Lewis index (*TLI*) and root mean squared error of approximation (*RMSEA*). CFI and TLI values above .90, and RMSEA below .08 were interpreted as acceptable fit (Hu & Bentler, 1999; Marsh et al., 2004). Next, we tested whether the measurement model was invariant across cultural contexts, using a multi‐group CFA to test configural invariance and metric invariance. ΔCFI ≤ .010, ΔRMSEA ≤ .015 and ΔSRMR ≤ .030 indicate metric invariance (Chen, 2007).

#### Hypothesis: Testing the associations between growth mindsets and the five broad social–emotional skills

Since students were nested in schools, we employed hierarchical linear modelling (HLM). A series of two‐level models with a maximum likelihood robust estimation approach was developed. Model 1 posited a growth mindset of social–emotional skills as the predictor variable. Model 2 added a growth mindset of intelligence as a student‐level covariate. Model 3 included gender and SES as student‐level covariates, as well as school climate, class size and school policies as school‐level predictors. Standardized coefficients were reported. *β*s above .05 are considered small but meaningful; those above .10 are considered moderate; and those above .25 are considered large (Keith, 2019, *p*. 62). The equations and the calculation of *pseudo* R‐squared are provided in the Supplementary Materials.

#### Supplementary analysis: Testing the role of growth mindsets on the 15 facet‐level social–emotional skills

We conducted HLM to determine the role of growth mindsets in predicting the 15 facet‐level skills. Due to space constraints, the information can be found in the supplementary materials.

#### Supplementary analysis in each of the 10 cities

We also conducted HLM to determine the role of growth mindsets across the 10 cities. Due to space constraints, the information can be found in the supplementary materials.

## RESULTS

### Preliminary analysis

Table 1 displays the descriptive analysis and correlations. The growth mindset of social–emotional skills and the growth mindset of intelligence were positively associated with social–emotional skills.

**TABLE 1:** Descriptive analysis and correlations.

	1	2	3	4	5	6	7	8	9	10	11	12
*Focal variable*												
Growth mindset of social–emotional skills	–											
*Social–emotional skills*												
Task performance	.12***	–										
Emotional regulation	.20***	.47***	–									
Engaging with others	.15***	.39***	.53***	–								
Collaboration	.17***	.54***	.46***	.53***	–							
Open‐mindedness	.11***	.51***	.36***	.48***	.48***	–						
*Student‐level covariates*												
Growth mindset of intelligence	.52***	.09***	.14***	.11***	.11***	.13***	–					
Gender	.03***	0	.24***	.12***	−.03***	−.05***	.03***	–				
SES	.16***	.04***	.05***	.09***	.12***	.03***	.13***	.02**	–			
*School‐level covariates*												
School climate	.18***	.32***	.47***	.56***	.47***	.30***	.14***	.07***	.08***	–		
Class size	−.12***	.03***	.01*	.07***	−.01*	.20***	−.11***	.01	−.28***	.05***	–	
School policy	.01	.01*	−.01	.01	.01	−.01	−.02**	.01	.02**	.01	.02**	–
Mean	6.41	1706.54	1575.01	1616.75	1721.76	1777.68	3.37	1.48	.2	45.86	5.34	49.53
*SD*	1.94	221.11	226.56	211.28	201.74	222.88	1.15	.5	1	12.07	2.57	2.3
Cronbach's alpha	.8	.89	.91	.89	.88	.89	n/a	n/a	n/a	.78	n/a	.67

*Note*: Female = 0, Male = 1.

Abbreviation: n/a, not applicable.

* *p* < .05.

** *p* < .01.

*** *p* < .001.

The construct validity results are presented in Table 2. Single‐group CFA was conducted for the five broad social–emotional skills, showing a good fit to the data in the overall sample and acceptable model fit indices: CFI > .90, TLI > .09 and RMSEA < .08.

**TABLE 2:** Model fit statistics for the five broad social–emotional skills.

	*χ* ^2^	CFI	TLI	RMSEA (90%CI)
Task performance	14,026***	.925	.910	.057 (.056, .058)
Emotional regulation	13,161***	.943	.919	.058 (.057, .059)
Engaging with others	10,646***	.952	.94	.048 (.047, .049)
Collaboration	9732***	.944	.929	.049 (.047, .050)
Open mindedness	10,190***	.938	.924	.049 (.048, .050)

*** *p* < .001.

The multi‐group CFA results are presented in Table 3. Fit indices showed good model‐data fit. Results indicated that configural invariance was achieved, which meant that the overall factor structure of social–emotional skills holds for all the samples across the 10 cities. Metric invariance was also achieved, which meant that the factor loadings were similar for the participants across the 10 cities.

**TABLE 3:** Multi‐group CFA invariance test.

	Chi‐squared	df	RMSEA	CFI	TLI	SRMR	Δ CFI	ΔRMSEA
*Task performance*								
Configural	17,852.42	1420	.06	.91	.9	.05	–	–
Metric	20,404.75	1564	.06	.9	.9	.06	.01	0
*Emotional regulation*								
Configural	17,461.77	1310	.06	.93	.9	.05	–	–
Metric	21,070.94	1463	.07	.92	.9	.07	.01	0
*Engaging with others*								
Configural	17,113.3	1530	.06	.93	.92	.05	–	–
Metric	20,253.54	1683	.06	.92	.91	.06	.01	0
*Collaboration*								
Configural	11,829	1340	.05	.94	.93	.04	–	–
Metric	13,540.42	1484	.05	.93	.92	.05	.01	0
*Openmindedness*								
Configural	13,913.28	1400	.06	.92	.91	.04	–	–
Metric	16,503.4	1544	.06	.91	.9	.06	.01	0

*Note*: Chi‐square statistics are all significant at *p* < .001. Configural invariance refers to the multi‐group comparison without imposing any equality constraints. Metric invariance refers to the comparison of the configural model with the model with imposed equality constraints at the factor loadings level.

### Hypothesis 1: Testing the associations between growth mindsets and the five broad social–emotional skills

Table 4 presents the HLM models for the five broad social–emotional skills. We calculated null‐model intra‐class correlations (ICCs). The ICCs range from .01 to .03. Although the ICCs are relatively small, given the nested nature of the data, we conducted multilevel analyses (Nezlek, 2008).

**TABLE 4:** Multilevel models predicting five broad social–emotional skills.

	Model 1					Model 2					Model 3				
	Task performance	Emotional regulation	Engaging with others	Collaboration	Open‐mindedness	Task performance	Emotional regulation	Engaging with others	Collaboration	Open‐mindedness	Task performance	Emotional regulation	Engaging with others	Collaboration	Open‐mindedness
*Focal variable*															
Growth mindset of social–emotional skills	.12***	.20***	.15***	.16**	.12***	.11***	.18***	.13***	.15***	.07***	.12***	.18***	.13***	.14***	.11***
*Student‐level covariates*															
Growth mindset of intelligence						.03***	.04***	.04***	.03***	.10***	.03***	.04***	.04***	.04***	.10***
Gender											.02*	.23***	.13***	−.03***	−.04***
SES											.07***	.03***	.12***	.09***	.12***
*School‐level covariates*															
Climate‐students' disruptive behaviour											.04**	0	.01	.02	.03*
Climate‐teachers' disruptive behaviour											−.01	−.03***	−.03**	.01	−.02
Class size											.07***	.05***	.11***	.03*	.27***
School policy											0	−.01	0	.02	−.01
*Random effects*															
Within‐school residual variance (*σ* ^2^)	.97	.95	.97	.96	.97	.97	.95	.97	.96	.96	.86	.71	.66	.71	.79
Between‐school variance (τ00, school)	.01	.01	.01	.02	.03	.01	.01	.01	.02	.03	.01	.01	.01	.01	.03
Variance attributable to between‐school variation (ICC school)	.01	.01	.01	.02	.03	.01	.01	.01	.02	.04	.01	.01	.01	.01	.03
Pseudo R2‐level 1	.02	.04	.02	.02	.01	.02	.04	.02	.02	.02	.06	.10	.08	.06	.13
Pseudo R2‐level 2	.02	−.04^a^	.08	.23	−.17	.03	−.04	.09	.24	−.19	.14	.57	.35	.76	.34

*Note*: Female = 0, Male = 1.

^a^ When a level‐1 predictor is added, the meaning of the level‐1 intercept changes and the variance of the level‐1 random intercept (i.e. the level‐2 variance component) could increase, resulting in a negative level‐2 pseudo R‐squared. However, this study mainly focused on predictive ability at student‐level (level‐1). And after adding all the covariates to model 3, the negative values were fixed.

* *p* < .05;

** *p* < .01;

*** *p* < .001.

#### Model 1: Growth mindset of social–emotional skills

Model 1 only included a growth mindset of social–emotional skills. Results showed that a growth mindset of social–emotional skills significantly predicted the five skills, with *β*s ranging from .12 (task performance and open‐mindedness) to .20 (emotional regulation).

#### Model 2: Growth mindsets of social–emotional skills and intelligence

Model 2 involved both the growth mindset of social–emotional skills and intelligence. The effect sizes of the mindset of social–emotional skills were smaller than those of Model 1, with *β*s ranging from .07 (open‐mindedness) to .18 (emotional regulation). The results showed that the effects of a growth mindset of social–emotional skills were larger than those found for intelligence mindsets.

#### Model 3: Student‐ and school‐level covariates

Model 3 added student‐level covariates and school‐level covariates. Growth mindsets of social–emotional skills were positively associated with social–emotional skills with smaller effect sizes compared with Model 1, with *β*s ranging from .11 (open‐mindedness) to .18 (emotional regulation).

### Supplementary analysis: Testing the role of growth mindsets on the 15 facet‐level social–emotional skills

The results of the 15 facet‐level skills are shown in Table S2. A growth mindset of social–emotional skills positively predicted 15 facet‐level skills with *β*s ranging from .02 (empathy) to .12 (emotional control). These positive associations were consistent with the five broad skills.

### Supplementary analysis in each of the 10 cities

We conducted a supplementary analysis for each of the 10 cities (Tables S3–S12). The results were largely consistent with what we found using the overall sample.

## DISCUSSION

This study aimed to explore the association between growth mindsets and social–emotional skills. The results indicated that a growth mindset of social–emotional skills is positively associated with social–emotional skills, with effect sizes larger than intelligence mindsets. This finding was consistent across the five broad, domain‐general skills and the 15 specific, facet‐level skills.

Our study extends the literature in several important ways. First, this study extended the current literature on social–emotional skills by investigating how motivational factors contribute to social–emotional flourishing (Collie & Martin, 2024). Past studies have devoted much attention to exploring the role of contextual factors (e.g. school‐wide curriculum; Jones et al., 2017) in fostering social–emotional skills. Despite the critical role of social contexts, the development of social–emotional skills includes both individual agency and environmental support (Collie & Martin, 2024). Our study is among the first to empirically investigate the effects of a growth mindset on diverse social–emotional skills. This finding aligns with past studies that indicated the critical role of growth mindsets in facilitating students' overall functioning (Dweck & Yeager, 2019; Yeager & Dweck, 2023). When students have growth mindsets, they exhibit greater effort in social–emotional tasks, pursue self‐development goals (Martin et al., 2019), and are more flexible in dealing with challenging social situations (Yeager et al., 2014). Accordingly, helping students feel that their social–emotional skills are malleable and changeable may also be important for their social–emotional functioning. Our study extends previous research by emphasizing the significance of growth mindsets in fostering social–emotional skills, offering a deeper understanding of the factors that facilitate its development.

Second, we also found that a growth mindset of social–emotional skills had a stronger association with social–emotional skills than intelligence mindsets. This finding is congruent with past research indicating that domain‐specific mindsets have a greater impact on outcomes within their respective domains (e.g. stress mindsets; Yeager et al., 2022). Although past studies revealed the importance of domain‐specific mindsets (Yeager et al., 2022), the current study extended these prior studies by focusing on the social–emotional domain. Hence, in understanding social–emotional skills, growth mindsets of social–emotional skills might be more relevant than other types of mindsets (e.g. emotion mindsets or intelligence mindsets).

Third, our analyses revealed consistent positive associations between growth mindsets and diverse social–emotional skills across 10 cities, showing the universal importance of growth mindsets in different cultures. Researchers have increasingly recognized the problems of drawing conclusions from research conducted disproportionately in one culture (Gelfand et al., 2017; King et al., 2018). This study is one of the few studies on social–emotional skills that draw on a broader range of countries across the globe.

Our study revealed medium effect sizes of focal variables on social–emotional skills, with *β*s ranging from .11 to .18 (Keith, 2019). This effect size is comparable to the impact of growth mindsets of intelligence in the academic domains (Burnette et al., 2023). Small to medium effect sizes are common in growth mindset interventions (Yeager & Dweck, 2020). For example, Sun et al. (2021) found a small effect size of growth mindsets on achievement among Chinese students (*d* ranging from .13 to .31). In another study, Kim and Park (2021) found that the associations between fixed mindsets and achievement were weak, with *r*s ranging from −.05 to −.22. Additionally, Sisk et al. (2018) conducted a meta‐analysis on 43 studies and found only a small effect size (*r* = .10) of growth mindset on academic outcomes.

It is worth noting that small to medium effect sizes are also typical in social–emotional skills interventions (Durlak et al., 2011). For example, Durlak et al. (2011) reviewed 213 school‐based social–emotional skills interventions and found small (Hedge's *g* = .22) to medium (Hedge's *g* = .57) effect sizes. Furthermore, many of the most common variables in educational and psychological research also have small effect sizes. Less than 3% of studies were found to be as large as *r* = .50 (Gignac & Szodorai, 2016). However, these effect sizes can have powerful downstream consequences (Yeager et al., 2019). When students repeatedly access, cultivate and deploy their growth mindsets over a prolonged period, these effects can compound over time (Götz et al., 2022). Consequently, the role of growth mindsets can persist over time and potentially influence students' longer‐term social–emotional trajectories.

### Theoretical and practical implications

Theoretically, our study contributes to existing research on both social–emotional skills and growth mindsets. First, our study highlights the importance of growth mindsets in social–emotional skills. Previous research has primarily emphasized the role of contextual factors in social–emotional skills. However, the process of developing social–emotional skills is inherently interactive, requiring both individual agency and supportive social contexts. Our study contributes to this body of work by highlighting the importance of growth mindsets in social–emotional skills, which have been relatively neglected in the social–emotional literature to date.

Second, our findings extend prior research on mindsets in social–emotional domains. Prior studies have examined mindsets of emotion (Ford & Gross, 2019), creativity (Ting & Yeh, 2024), and stress (Yeager et al., 2022) separately. Our study simultaneously investigated the effects of growth mindsets on a wide range of social–emotional skills, contributing to a comprehensive understanding of the role of mindsets in various social–emotional skills.

Third, our study also adds to cross‐cultural generalizability. We conducted analyses across the 10 cities. This demonstrates that endorsing a growth mindset of social–emotional skills is important across diverse cultural contexts.

Practically, our study provides implications for educators to help their students develop social–emotional skills. Prior studies have indicated that relatively simple and cost‐effective growth mindset interventions can have long‐lasting effects (Yeager et al., 2019). For example, growth mindset interventions have found that relatively minimal intervention dosage (less than one hour) was sufficient to improve the relative outcomes (e.g. Yeager et al., 2022). Teachers can help students reflect on instances they have developed their social–emotional skills after struggling in challenging situations and how they might apply a growth mindset to develop their social–emotional skills (Aronson et al., 2002; Yeager & Dweck, 2020).

Compared to relatively stable personal traits, social–emotional skills are malleable and can be changed through school‐based interventions. Social skills mindsets (e.g. aggressiveness) and emotion mindsets interventions have provided promising approaches for cultivating students' social–emotional skills (Ford & Gross, 2018; Yeager et al., 2013). For example, social skills mindsets workshops (e.g. teaching the idea that people have the potential to improve their social skills and providing opportunities to practice these social skills) improved students' social skills (e.g. reduced aggressiveness; Yeager et al., 2013). Emotion mindsets intervention could enhance students' adaptive emotion mindsets and proactive emotional regulation, such as teaching emotion malleability, asking them to summarize what they had learned from the lesson and requiring students to do a self‐persuasion exercise (Rudolph et al., 2025). When students hold growth mindsets about social–emotional skills, they are more proactive in social interactions and internal regulation, thereby facilitating the effective development of these skills.

## LIMITATIONS AND FUTURE DIRECTIONS

Some limitations need to be acknowledged. First, the cross‐sectional nature of our data prevents us from drawing causal relationships. A substantial body of work has established growth mindset as a predictor of focal outcomes (e.g. Burnette et al., 2023). However, we recognize that the cross‐sectional data limit causal claims. Longitudinal or experimental designs are recommended to explore causal relationships. Second, the current study is based on 15‐year‐old students spread across 10 cities. Although the SSES dataset included 10 cities across nine countries, the results may not be generalizable to other ages or countries. We encourage future studies to triangulate our research findings with those from other age groups and countries. Third, this study relied on self‐report surveys. Although the use of self‐reports is quite common (Guo et al., 2023; Kankaraš & Suarez‐Alvarez, 2019; Wang & King, 2025), we suggest that future studies triangulate our results with other sources of data such as qualitative interviews, behavioural measures, and informant ratings. Fourth, this study is a secondary analysis of the OECD's SSES database. We encourage future studies to include academic outcomes to test the full effects of domain‐specific and general mindsets. Fifth, there may be other factors (e.g. emotion regulation strategies) involved in the relationship between growth mindsets and social–emotional skills. Uncovering these mediators can significantly enhance our theoretical understanding of why and how mindsets of social–emotional skills might be associated with the skills themselves. Although we are not yet aware of specific studies that have been done, some potential mediators might include attribution styles, social goals, emotion regulation techniques and social regulation strategies among others. We encourage future studies to examine potential mediators in growth mindsets of social–emotional skills and outcomes.

## CONCLUSION

Our study contributes to both the mindsets literature and social–emotional skills literature by revealing the important role of growth mindsets in the social–emotional domain. Students who believe their social–emotional skills are malleable demonstrate the highest levels of social–emotional skills. This study extends prior research, which has mostly focused on the role of intelligence mindsets on academic outcomes. It seems that researchers may need to expand their theoretical purview beyond intelligence mindsets and be cognizant of the critical role played by students' social‐emotional mindsets.

## AUTHOR CONTRIBUTIONS

**Jianhua Zhang:** Conceptualization; methodology; software; data curation; formal analysis; validation; visualization; writing – review and editing; writing – original draft. **Faming Wang:** Conceptualization; writing – review and editing. **Ronnel B. King:** Conceptualization; writing – review and editing; supervision.

## FUNDING INFORMATION

This work was supported by the Research Grants Council of Hong Kong under the General Research Fund (GRF) [498575193].

## CONFLICT OF INTEREST STATEMENT

The authors have no conflict of interest to declare.

## ETHICS STATEMENT

This study conducts a secondary analysis using openly available OECD‐SSES data. The authors did not directly employ any intervention on human subjects.

## Supporting information


**Table S1.** Description of variables.
**Table S2.** Multilevel Models Predicting 15 Specific Facet‐Level Social–Emotional Skills.
**Table S3.** Multilevel Models Predicting Social–Emotional Skills (Ottawa).
**Table S4.** Multilevel Models Predicting Social–Emotional Skills (Houston).
**Table S5.** Multilevel Models Predicting Social–Emotional Skills (Bogota).
**Table S6.** Multilevel Models Predicting Social–Emotional Skills (Manizales).
**Table S7.** Multilevel Models Predicting Social–Emotional Skills (Helsinki).
**Table S8.** Multilevel Models Predicting Social–Emotional Skills (Moscow).
**Table S9.** Multilevel Models Predicting Social–Emotional Skills (Istanbul).
**Table S10.** Multilevel Models Predicting Social–Emotional Skills (Daegu).
**Table S11.** Multilevel Models Predicting Social–Emotional Skills (Sintra).
**Table S12.** Multilevel Models Predicting Social–Emotional Skills (Suzhou).
**Table S13.** Model Fit of Bifactor CFA Models.

## Data Availability

The data that support the findings of this study are openly available at the OECD website (https://www.oecd.org/education/ceri/social‐emotional‐skills‐study/data.htm).

## Footnotes

^1^
The OECD technical report analysed item dimensionality and measurement invariance for the final scaling through two steps. First, the OECD analysed measurement invariance with multiple‐group confirmatory factor analysis across age, gender and cities. Second, the OECD scaled items and generated scores for participants through Item Response Theory Generalized Partial Credit Model.

^2^
This item was consistent with the original growth mindsets questionnaires (Dweck & Leggett, 1988). The items were recoded to represent students' growth mindsets (Bernardo et al., 2021).

^3^
SES was calculated based on students' home possessions, parents' highest occupational status and parents' highest level of education.
