# Supplementary material for: Beyond intelligence: Exploring the role of growth mindsets in the domain of social–emotional skills
Source: Br J Educ Psychol. 2025 Oct 2;96(2):539–57. doi: 10.1111/bjep.70037 (PMC13155037; doi:10.1111/bjep.70037)
Supplement: Supplementary file 1 — Table S1. Description of variables. Table S2. Multilevel Models Predicting 15 Specific Facet‐Level Social–Emotional Skills. Table S3. Multilevel Models Predicting Social–Emotional Skills (Ottawa). Table S4. Multilevel Models Predicting Social–Emotional Skills (Houston). Table S5. Multilevel Models Predicting Social–Emotional Skills (Bogota). Table S6. Multilevel Models Predicting Social–Emotional Skills (Manizales). Table S7. Multilevel Models Predicting Social–Emotional Skills (Helsinki). Table S8. Multilevel Models Predicting Social–Emotional Skills (Moscow). Table S9. Multilevel Models Predicting Social–Emotional Skills (Istanbul). Table S10. Multilevel Models Predicting Social–Emotional Skills (Daegu). Table S11. Multilevel Models Predicting Social–Emotional Skills (Sintra). Table S12. Multilevel Models Predicting Social–Emotional Skills (Suzhou). Table S13. Model Fit of Bifactor CFA Models. [file BJEP-96-539-s001.docx]

**Supplementary material**

**Section A**

**Table S1**. Description of variables

| **Variables** | **Alpha** | **Omega** | **Item example** | **Scale range** |
| --- | --- | --- | --- | --- |
| **Social and emotional skills** |  |  |  |  |
| Task performance | 0.89 | 0.89 | N/A | N/A |
| Emotional regulation | 0.91 | 0.91 | N/A | N/A |
| Engaging with others | 0.89 | 0.88 | N/A | N/A |
| Collaboration | 0.88 | 0.88 | N/A | N/A |
| Open mindedness | 0.89 | 0.89 | N/A | N/A |
| Assertiveness | 0.90 | 0.90 | Likes to be a leader in their class | 1 Strongly disagree 2 Disagree 3 Neither agree nor disagree 4 Agree 5 Strongly agree |
| Cooperation | 0.78 | 0.80 | Like to help others | 1 Strongly disagree 2 Disagree 3 Neither agree nor disagree 4 Agree 5 Strongly agree |
| Creativity | 0.79 | 0.79 | Original, come up with new ideas | 1 Strongly disagree 2 Disagree 3 Neither agree nor disagree 4 Agree 5 Strongly agree |
| Curiosity | 0.80 | 0.81 | Like learning new things | 1 Strongly disagree 2 Disagree 3 Neither agree nor disagree 4 Agree 5 Strongly agree |
| Emotional control | 0.81 | 0.81 | Keep [their] emotions under control | 1 Strongly disagree 2 Disagree 3 Neither agree nor disagree 4 Agree 5 Strongly agree |
| Empathy | 0.74 | 0.74 | Important to [them] that [their] friends are okay | 1 Strongly disagree 2 Disagree 3 Neither agree nor disagree 4 Agree 5 Strongly agree |
| Energy | 0.78 | 0.79 | Full of energy | 1 Strongly disagree 2 Disagree 3 Neither agree nor disagree 4 Agree 5 Strongly agree |
| Optimism | 0.86 | 0.86 | Always positive about the future | 1 Strongly disagree 2 Disagree 3 Neither agree nor disagree 4 Agree 5 Strongly agree |
| Persistence | 0.83 | 0.84 | Keep working on a task until it is finished | 1 Strongly disagree 2 Disagree 3 Neither agree nor disagree 4 Agree 5 Strongly agree |
| Responsibility | 0.76 | 0.76 | Reliable and can always be counted on | 1 Strongly disagree 2 Disagree 3 Neither agree nor disagree 4 Agree 5 Strongly agree |
| Self-control | 0.76 | 0.76 | Can control [their] actions | 1 Strongly disagree 2 Disagree 3 Neither agree nor disagree 4 Agree 5 Strongly agree |
| Sociability | 0.81 | 0.81 | Have many friends | 1 Strongly disagree 2 Disagree 3 Neither agree nor disagree 4 Agree 5 Strongly agree |
| Stress-resistance | 0.86 | 0.86 | Worry about many things (R) | 1 Strongly disagree 2 Disagree 3 Neither agree nor disagree 4 Agree 5 Strongly agree |
| Tolerance | 0.80 | 0.81 | Ask questions about other cultures | 1 Strongly disagree 2 Disagree 3 Neither agree nor disagree 4 Agree 5 Strongly agree |
| Trust | 0.84 | 0.85 | Think most of [their] classmates keep their promises | 1 Strongly disagree 2 Disagree 3 Neither agree nor disagree 4 Agree 5 Strongly agree |
|  |  |  |  |  |

**Section B**

**Section B: The equations.**

Level 1:

*Yij* = *β0j* + *β1(growth mindset of social-emotional skills)ij* + …+ *βjXij* + *rij*

Level 2:

*β0j* = *γ00*+*γ01Gj* +…+*γ0jGj*+u0j

*β1* = *γ10*

…

*Βj=γj0*

In this formula, *Yij* represents the *i*th student’s social-emotional skills in school *j*; *Xij* represents the value of predictor for *i*th student in the *j*th school; *β0j* represents the intercept of school *j*; *β1*-*βj* represents the fixed effects of the top variables; *rij* represents the residuals at the student level; *Gj* represents the value of *j*th school-level predictor; *γ0j* represents the slope for the *j*th school-level predictor; *u0j* represents the residuals at the school level.

In the literature, there were several approaches to estimate the effect sizes of HLM models (e.g., R-squared measure). One of the approaches was proposed by Raudenbush and Bryk (1992), which was referred to as *pseudo* R-squared. The approach is variance-component-specific and computes an R-squared statistic for each variance component in a model. Level 1 *pseudo* R-squared determined the relative importance of the Level-1 predictors in explaining the within-cluster variance. Level 2 *pseudo* R-squared determined the relative importance of the Level-2 predictors in explaining the variance of the random cluster effects.

**Section C**

**Table S2**.

*Multilevel Models Predicting 15 Specific Facet-Level Social-Emotional Skills*

|  | **Task performance** | | | **Emotional regulation** | | | **Engaging with others** | | | **Collaboration** | | | **Open-mindedness** | | |
| --- | --- | --- | --- | --- | --- | --- | --- | --- | --- | --- | --- | --- | --- | --- | --- |
|  | Self-control | Responsibility | Persistence | Stress-resistance | Emotional-control | Optimism | Energy | Assertiveness | Sociability | Empathy | Cooperation | Trust | Curiosity | Creativity | Tolerance |
| **Focal variable** |  |  |  |  |  |  |  |  |  |  |  |  |  |  |  |
| Growth mindset of social-emotional skills | 0.09*** | 0.10*** | 0.08*** | 0.13*** | 0.15*** | 0.15*** | 0.10*** | 0.07*** | 0.12*** | 0.07** | 0.09*** | 0.11*** | 0.07*** | 0.08*** | 0.08*** |
| ***Student-level covariates*** |  |  |  |  |  |  |  |  |  |  |  |  |  |  |  |
| Growth mindset of intelligence | 0.03*** | 0.02** | 0.04*** | 0.01 | 0.03*** | 0.05*** | 0.04*** | 0.04*** | 0.01 | 0.05*** | 0.04*** | 0.01 | 0.10*** | 0.08*** | 0.07*** |
| Gender | 0.05*** | -0.07*** | 0.02** | 0.26*** | 0.20*** | 0.10*** | 0.15*** | 0.05*** | 0.09*** | -0.09*** | -0.08*** | 0.08*** | -0.01* | 0.05*** | -0.14*** |
| SES | 0.02* | 0.04*** | 0.04*** | 0 | 0.05*** | 0.03*** | 0.02** | 0.15*** | 0.08*** | 0.09*** | 0.03** | 0.04*** | 0.05*** | 0.13*** | 0.09*** |
| ***School-level covariates*** |  |  |  |  |  |  |  |  |  |  |  |  |  |  |  |
| Climate-students’ disruptive behavior | -0.03* | -0.03 | -0.01 | 0 | 0.01 | 0.01 | -0.01 | -0.01 | 0 | -0.05** | -0.02 | -0.04* | 0 | -0.01 | -0.02 |
| Climate-teachers’ disruptive behavior | -0.02 | 0.02 | -0.02 | -0.06*** | 0 | 0.02 | -0.03 | 0.01 | -0.03** | -0.02 | -0.01 | 0.06** | -0.01 | -0.03 | 0.03 |
| Class size | 0.06*** | 0.08*** | 0.10*** | 0.09*** | -0.07*** | 0.12*** | 0.14*** | 0.12*** | 0.06*** | 0.05** | 0.14*** | -0.09*** | 0.31*** | 0.20*** | 0.21*** |
| School policy | 0.01 | 0.01 | 0 | -0.01 | -0.02 | 0 | -0.02 | 0.02 | 0 | 0 | -0.01 | 0 | -0.01 | -0.01 | 0.01 |
| **Random effects** |  |  |  |  |  |  |  |  |  |  |  |  |  |  |  |
| Within-school residual variance (σ2) | 0.95 | 0.97 | 0.96 | 0.90 | 0.90 | 0.91 | 0.92 | 0.92 | 0.96 | 0.93 | 0.93 | 0.89 | 0.85 | 0.92 | 0.89 |
| Between-school variance (τ00, school) | 0.01 | 0.01 | 0.01 | 0.01 | 0.01 | 0.01 | 0.01 | 0.01 | 0.00 | 0.01 | 0.01 | 0.01 | 0.02 | 0.01 | 0.02 |
| Variance attributable to between-school variation (ICC school) | 0.01 | 0.01 | 0.01 | 0.01 | 0.01 | 0.01 | 0.01 | 0.01 | 0.01 | 0.01 | 0.01 | 0.01 | 0.02 | 0.01 | 0.02 |
| Pseudo R2-level 1 | 0.04 | 0.02 | 0.04 | 0.09 | 0.09 | 0.07 | 0.06 | 0.07 | 0.03 | 0.05 | 0.06 | 0.08 | 0.13 | 0.07 | 0.09 |
| Pseudo R2-level 2 | 0.20 | -0.09 | 0.14 | 0.22 | 0.49 | 0.53 | 0.19 | 0.39 | 0.53 | 0.69 | 0.41 | 0.75 | 0.52 | 0.23 | 0.17 |

***Note****.* Female=0, Male=1. **p* < 0.05; ***p* < 0.01; ****p* < 0.001.

**Table S3.**

*Multilevel Models Predicting Social-emotional Skills (Ottawa)*

|  | Task performance | Emotional regulation | Engaging with others | Collaboration | Open-mindedness |
| --- | --- | --- | --- | --- | --- |
| **Fixed effect** |  |  |  |  |  |
| **Student-level** |  |  |  |  |  |
| Growth mindset about social-emotional skills | 0.16*** | 0.21*** | 0.12*** | 0.08** | 0.07* |
| Growth mindset about intelligence | 0.07** | 0.06* | 0.02 | 0.07* | 0.15*** |
| Gender | -0.13** | 0.48*** | 0.18*** | -0.13** | -0.15** |
| SES | 0.11*** | 0.04 | 0.13*** | 0.13*** | 0.15*** |
| **School-level** |  |  |  |  |  |
| Climate-students’ disruptive behavior | -0.02 | -0.01 | 0.04 | -0.02 | -0.02 |
| Climate-teachers’ disruptive behavior | 0.02 | -0.04 | -0.03 | 0.02 | 0.04 |
| Class size | 0.03 | -0.03 | 0 | 0 | 0.06 |
| School policy | -0.01 | 0.06* | 0.01 | -0.02 | 0.03 |
| **Random effects** |  |  |  |  |  |
| Within-school residual variance (σ2) | 0.89 | 0.88 | 0.92 | 0.92 | 0.86 |
| Between-school variance (τ00, school) | 0.00 | 0.00 | 0.01 | 0.00 | 0.02 |
| Variance attributable to between-school variation (ICC school) | 0 | 0 | 0.01 | 0 | 0.03 |
| Pseudo$R^{2}$-Level1 | 0.10 | 0.12 | 0.07 | 0.07 | 0.11 |
| Pseudo$R^{2}$-Level2 | 1.00 | 0.81 | 0.63 | 1.00 | 0.35 |

**Table S4.**

*Multilevel Models Predicting Social-emotional Skills (Houston)*

|  | Task performance | Emotional regulation | Engaging with others | Collaboration | Open-mindedness |
| --- | --- | --- | --- | --- | --- |
| **Fixed effect** |  |  |  |  |  |
| **Student-level** |  |  |  |  |  |
| Growth mindset about social-emotional skills | 0.13*** | 0.25*** | 0.18*** | 0.15*** | 0.14*** |
| Growth mindset about intelligence | 0.02 | -0.03 | -0.01 | 0.02 | 0.08*** |
| Gender | -0.12** | 0.47*** | 0.27*** | -0.04 | -0.21*** |
| SES | 0.12*** | 0.07*** | 0.17*** | 0.12*** | 0.16*** |
| **School-level** |  |  |  |  |  |
| Climate-students’ disruptive behavior | 0.06* | 0.07** | 0.06* | -0.07* | 0.04 |
| Climate-teachers’ disruptive behavior | -0.03 | -0.04 | -0.06* | -0.01 | -0.05* |
| Class size | 0.01 | 0.01 | -0.03 | -0.02 | -0.04 |
| School policy | 0.03 | -0.01 | 0 | -0.01 | 0 |
| **Random effects** |  |  |  |  |  |
| Within-school residual variance (σ2) | 0.95 | 0.86 | 0.91 | 0.92 | 0.90 |
| Between-school variance (τ00, school) | 0.00 | 0.01 | 0.01 | 0.00 | 0.00 |
| Variance attributable to between-school variation (ICC school) | 0 | 0.01 | 0.01 | 0 | 0 |
| Pseudo$R^{2}$-Level1 | 0.05 | 0.14 | 0.09 | 0.05 | 0.08 |
| Pseudo$R^{2}$-Level2 | 1.00 | -3.17 | -0.16 | 1.00 | 1.00 |

**Table S5.**

*Multilevel Models Predicting Social-emotional Skills (Bogota)*

|  | Task performance | Emotional regulation | Engaging with others | Collaboration | Open-mindedness |
| --- | --- | --- | --- | --- | --- |
| **Fixed effect** |  |  |  |  |  |
| **Student-level** |  |  |  |  |  |
| Growth mindset about social-emotional skills | 0.13*** | 0.22*** | 0.16*** | 0.10*** | 0.05* |
| Growth mindset about intelligence | 0.01 | -0.03 | -0.02 | 0.02 | 0.13*** |
| Gender | -0.05 | 0.48*** | 0.28*** | -0.11** | -0.17*** |
| SES | 0.05* | 0.01 | 0.12*** | 0.11*** | 0.14*** |
| **School-level** |  |  |  |  |  |
| Climate-students’ disruptive behavior | 0.01 | 0 | -0.04 | -0.01 | -0.01 |
| Climate-teachers’ disruptive behavior | -0.02 | 0.03 | 0 | -0.01 | 0.02 |
| Class size | -0.03 | 0 | 0 | -0.02 | 0.01 |
| School policy | 0.01 | 0.01 | 0 | 0.04 | 0.02 |
| **Random effects** |  |  |  |  |  |
| Within-school residual variance (σ2) | 0.96 | 0.88 | 0.92 | 0.94 | 0.93 |
| Between-school variance (τ00, school) | 0.01 | 0.01 | 0.00 | 0.00 | 0.00 |
| Variance attributable to between-school variation (ICC school) | 0.01 | 0.01 | 0 | 0 | 0 |
| Pseudo$R^{2}$-Level1 | 0.02 | 0.10 | 0.06 | 0.02 | 0.03 |
| Pseudo$R^{2}$-Level2 | 0.46 | 0.42 | 0.86 | 0.97 | 0.94 |

**Table S6.**

*Multilevel Models Predicting Social-emotional Skills (Manizales)*

|  | Task performance | Emotional regulation | Engaging with others | Collaboration | Open-mindedness |
| --- | --- | --- | --- | --- | --- |
| **Fixed effect** |  |  |  |  |  |
| **Student-level** |  |  |  |  |  |
| Growth mindset about social- emotional skills | 0.16*** | 0.27*** | 0.20*** | 0.18*** | 0.09** |
| Growth mindset about intelligence | -0.02 | -0.09** | -0.04 | -0.04 | 0.11*** |
| Gender | -0.09 | 0.51*** | 0.29*** | -0.16** | -0.19*** |
| SES | 0.05 | 0.06* | 0.13*** | 0.10*** | 0.16*** |
| **School-level** |  |  |  |  |  |
| Climate-students’ disruptive behavior | -0.02 | 0.04 | 0 | -0.07* | -0.03 |
| Climate-teachers’ disruptive behavior | 0.04 | 0.05 | 0.04 | 0.02 | 0.01 |
| Class size | 0 | 0.04 | 0.03 | 0.02 | 0.02 |
| School policy | 0.02 | 0.06 | 0.02 | 0.01 | -0.01 |
| **Random effects** |  |  |  |  |  |
| Within-school residual variance (σ2) | 0.9803206 | 0.8909619 | 0.9562205 | **0.9799074** | 0.9307837 |
| Between-school variance (τ00, school) | 0.0060237 | 0.0072346 | 0 | 0 | 0 |
| Variance attributable to between-school variation (ICC school) | 0.01 | 0.01 | 0 | 0 | 1 |
| Pseudo$R^{2}$-Level1 | 0.01 | 0.10 | 0.03 | 0.00 | 0.04 |
| Pseudo$R^{2}$-Level2 | 0.41 | 0.48 | 1.00 | 1.00 | 1.00 |

**Table S7.**

*Multilevel Models Predicting Social-emotional Skills (Helsinki)*

|  | Task performance | Emotional regulation | Engaging with others | Collaboration | Open-mindedness |
| --- | --- | --- | --- | --- | --- |
| **Fixed effect** |  |  |  |  |  |
| **Student-level** |  |  |  |  |  |
| Growth mindset about social- emotional skills | 0.12*** | 0.12*** | 0.12*** | 0.12*** | 0.13*** |
| Growth mindset about intelligence | 0.08** | 0.07** | 0.05* | 0.09** | 0.15*** |
| Gender | -0.06 | 0.69*** | 0.35*** | -0.25*** | -0.13** |
| SES | 0.11*** | -0.01 | 0.09*** | 0.11*** | 0.15*** |
| **School-level** |  |  |  |  |  |
| Climate-students’ disruptive behavior | -0.01 | -0.03 | 0.02 | -0.04 | -0.02 |
| Climate-teachers’ disruptive behavior | 0.01 | 0 | 0.02 | 0.03 | -0.01 |
| Class size | -0.05 | -0.04 | -0.06 | 0.03 | 0.07* |
| School policy | 0.04 | 0.04 | 0.02 | 0.01 | 0.02 |
| **Random effects** |  |  |  |  |  |
| Within-school residual variance (σ2) | 0.94 | 0.84 | 0.92 | 0.88 | 0.82 |
| Between-school variance (τ00, school) | 0.00 | 0.00 | 0.00 | 0.00 | 0.01 |
| Variance attributable to between-school variation (ICC school) | 0 | 0 | 0 | 0 | 0.01 |
| Pseudo$R^{2}$-Level1 | 0.05 | 0.14 | 0.06 | 0.10 | 0.12 |
| Pseudo$R^{2}$-Level2 | 1.00 | 1.00 | 0.77 | 1.00 | 0.89 |

**Table S8.**

*Multilevel Models Predicting Social-emotional Skills (Moscow)*

|  | Task performance | Emotional regulation | Engaging with others | Collaboration | Open-mindedness |
| --- | --- | --- | --- | --- | --- |
| **Fixed effect** |  |  |  |  |  |
| **Student-level** |  |  |  |  |  |
| Growth mindset about social- emotional skills | 0.11*** | 0.18*** | 0.12*** | 0.12*** | 0.10*** |
| Growth mindset about intelligence | 0.04*** | 0.04*** | 0.04*** | 0.05*** | 0.10*** |
| Gender | 0 | 0.48*** | 0.24*** | -0.08*** | -0.10*** |
| SES | 0.04*** | 0.03*** | 0.11*** | 0.07*** | 0.12*** |
| **School-level** |  |  |  |  |  |
| Climate-students’ disruptive behavior | -0.03 | 0.01 | -0.01 | -0.05** | -0.01 |
| Climate-teachers’ disruptive behavior | -0.01 | -0.02 | -0.02 | 0.02 | 0 |
| Class size | 0.10*** | 0.05*** | 0.14*** | 0.04** | 0.30*** |
| School policy | 0.01 | -0.01 | 0 | 0 | 0 |
| **Random effects** |  |  |  |  |  |
| Within-school residual variance (σ2) | 0.95 | 0.90 | 0.93 | 0.93 | 0.86 |
| Between-school variance (τ00, school) | 0.01 | 0.01 | 0.01 | 0.01 | 0.02 |
| Variance attributable to between-school variation (ICC school) | 0.01 | 0.01 | 0.01 | 0.01 | 0.02 |
| Pseudo$R^{2}$-Level1 | 0.04 | 0.09 | 0.06 | 0.05 | 0.12 |
| Pseudo$R^{2}$-Level2 | 0.10 | 0.36 | 0.16 | 0.79 | 0.30 |

**Table S9.**

*Multilevel Models Predicting Social-emotional Skills (Istanbul)*

|  | Task performance | Emotional regulation | Engaging with others | Collaboration | Open-mindedness |
| --- | --- | --- | --- | --- | --- |
| **Fixed effect** |  |  |  |  |  |
| **Student-level** |  |  |  |  |  |
| Growth mindset about social- emotional skills | -0.07** | 0.05* | -0.01 | -0.07** | -0.02 |
| Growth mindset about intelligence | 0.07*** | 0.05* | 0.05* | 0.10*** | 0.12*** |
| Gender | 0.09* | 0.48*** | 0.30*** | -0.04 | -0.05 |
| SES | -0.04* | 0.03 | 0.08*** | 0 | 0.12*** |
| **School-level** |  |  |  |  |  |
| Climate-students’ disruptive behavior | 0.01 | 0.04 | 0.02 | -0.03 | -0.04 |
| Climate-teachers’ disruptive behavior | -0.03 | -0.04 | 0 | -0.01 | 0.01 |
| Class size | 0.01 | 0.02 | 0.02 | 0.01 | 0 |
| School policy | -0.01 | -0.01 | -0.01 | -0.03 | 0 |
| **Random effects** |  |  |  |  |  |
| Within-school residual variance (σ2) | 0.99 | 0.92 | 0.97 | 0.99 | 0.95 |
| Between-school variance (τ00, school) | 0.00 | 0.01 | 0.00 | 0.00 | 0.01 |
| Variance attributable to between-school variation (ICC school) | 0 | 0.01 | 0 | 0 | 0.01 |
| Pseudo$R^{2}$-Level1 | 0.00 | 0.05 | 0.02 | 0.00 | 0.03 |
| Pseudo$R^{2}$-Level2 | 0.49 | 0.48 | 1.00 | 1.00 | 0.35 |

**Table S10.**

*Multilevel Models Predicting Social-emotional Skills (Daego)*

|  | Task performance | Emotional regulation | Engaging with others | Collaboration | Open-mindedness |
| --- | --- | --- | --- | --- | --- |
| **Fixed effect** |  |  |  |  |  |
| **Student-level** |  |  |  |  |  |
| Growth mindset about social- emotional skills | 0.22*** | 0.25*** | 0.22*** | 0.22*** | 0.24*** |
| Growth mindset about intelligence | 0.13*** | 0.17*** | 0.13*** | 0.13*** | 0.15*** |
| Gender | 0.06 | 0.36*** | 0.10** | 0.03 | 0.12** |
| SES | 0.07*** | 0.07*** | 0.13*** | 0.06** | 0.14*** |
| **School-level** |  |  |  |  |  |
| Climate-students’ disruptive behavior | 0.01 | -0.01 | -0.02 | 0 | -0.02 |
| Climate-teachers’ disruptive behavior | -0.02 | -0.01 | -0.01 | -0.03 | -0.04* |
| Class size | -0.01 | -0.01 | 0.04 | 0.01 | -0.03 |
| School policy | -0.04 | -0.03 | -0.04 | -0.08** | -0.07** |
| **Random effects** |  |  |  |  |  |
| Within-school residual variance (σ2) | 0.89 | 0.81 | 0.87 | 0.86 | 0.82 |
| Between-school variance (τ00, school) | 0.01 | 0.01 | 0.00 | 0.01 | 0.00 |
| Variance attributable to between-school variation (ICC school) | 0.01 | 0.01 | 0.01 | 0.02 | 0.01 |
| Pseudo$R^{2}$-Level1 | 0.09 | 0.15 | 0.10 | 0.11 | 0.16 |
| Pseudo$R^{2}$-Level2 | 0.64 | 0.73 | 0.84 | 0.57 | 0.85 |

**Table S11.**

*Multilevel Models Predicting Social-emotional Skills (Sintra)*

|  | Task performance | Emotional regulation | Engaging with others | Collaboration | Open-mindedness |
| --- | --- | --- | --- | --- | --- |
| **Fixed effect** |  |  |  |  |  |
| **Student-level** |  |  |  |  |  |
| Growth mindset about social- emotional skills | 0.10** | 0.21*** | 0.10** | 0.08* | 0.11*** |
| Growth mindset about intelligence | 0 | 0.01 | -0.01 | 0.01 | 0.01 |
| Gender | -0.18** | 0.49*** | 0.28*** | -0.20*** | -0.28*** |
| SES | 0.13*** | 0.05* | 0.13*** | 0.08** | 0.13*** |
| **School-level** |  |  |  |  |  |
| Climate-students’ disruptive behavior | -0.03 | 0.03 | 0.02 | -0.03 | 0.02 |
| Climate-teachers’ disruptive behavior | 0.10** | 0.05 | 0.01 | 0.07 | 0.07* |
| Class size | 0.12*** | 0.01 | 0.04 | 0.14*** | 0.09** |
| School policy | 0.03 | 0.01 | 0 | 0.02 | 0.01 |
| **Random effects** |  |  |  |  |  |
| Within-school residual variance (σ2) | 0.93 | 0.89 | 0.95 | 0.96 | 0.89 |
| Between-school variance (τ00, school) | 0.00 | 0.01 | 0.00 | 0.00 | 0.00 |
| Variance attributable to between-school variation (ICC school) | 0 | 0.01 | 0 | 0 | 0 |
| Pseudo$R^{2}$-Level1 | 0.05 | 0.10 | 0.05 | 0.01 | 0.10 |
| Pseudo$R^{2}$-Level2 | 1.00 | -0.77 | 1.00 | 1.00 | 1.00 |

**Table S12.**

*Multilevel Models Predicting Social-emotional Skills (Suzhou)*

|  | Task performance | Emotional regulation | Engaging with others | Collaboration | Open-mindedness |
| --- | --- | --- | --- | --- | --- |
| **Fixed effect** |  |  |  |  |  |
| **Student-level** |  |  |  |  |  |
| Growth mindset about social- emotional skills | 0.15*** | 0.19*** | 0.21*** | 0.19*** | 0.22*** |
| Growth mindset about intelligence | 0.12*** | 0.12*** | 0.11*** | 0.10*** | 0.11*** |
| Gender | 0.16*** | 0.28*** | 0.16*** | 0.05 | 0.06* |
| SES | 0.10*** | 0.09*** | 0.15*** | 0.08*** | 0.16*** |
| **School-level** |  |  |  |  |  |
| Climate-students’ disruptive behavior | -0.15*** | -0.07* | -0.02 | -0.09** | -0.13*** |
| Climate-teachers’ disruptive behavior | 0.11** | 0.05 | 0.02 | 0.09** | 0.09* |
| Class size | 0 | -0.02 | -0.02 | 0.01 | 0.05* |
| School policy | 0.01 | -0.03 | -0.04 | -0.01 | -0.03 |
| **Random effects** |  |  |  |  |  |
| Within-school residual variance (σ2) | 0.90 | 0.88 | 0.86 | 0.90 | 0.85 |
| Between-school variance (τ00, school) | 0 | 0 | 0 | 0 | 0 |
| Variance attributable to between-school variation (ICC school) | 0 | 0 | 0 | 0 | 0 |
| Pseudo$R^{2}$-Level1 | 0.07 | 0.10 | 0.13 | 0.08 | 0.11 |
| Pseudo$R^{2}$-Level2 | 1.00 | 1.00 | 1.00 | 1.00 | 1.00 |

**Section D**

**Table S13.**

*Model Fit of Bifactor CFA Models*

|  | χ2(df) | AIC | CFI | RMSEA | SRMR |
| --- | --- | --- | --- | --- | --- |
| Task performance | 29.90*** | 1282295.45 | 0.983 | 0.031 | 0.016 |
| Emotional regulation | 942.72*** | 1589722.54 | 0.937 | 0.056 | 0.042 |
| Engaging with others | 915.92*** | 1495642.57 | 0.971 | 0.039 | 0.041 |
| Collaboration | 65.65*** | 1337966.61 | 0.952 | 0.047 | 0.046 |
| Open-mindedness | 62.05*** | 1366061.45 | 0.935 | 0.045 | 0.031 |
